# Safe Stop IPI-NIVO trial: early discontinuation of nivolumab upon achieving a complete or partial response in patients with irresectable stage III or metastatic melanoma treated with first-line ipilimumab-nivolumab – study protocol

**DOI:** 10.1186/s12885-024-12336-0

**Published:** 2024-05-23

**Authors:** J. C. Janssen, B. van Dijk, K. de Joode, M. J. B. Aarts, F. W. P. J. van den Berkmortel, C. U. Blank, M. J. Boers-Sonderen, A. J. M. van den Eertwegh, J. W. B. de Groot, M. Jalving, M. J. A. de Jonge, A. Joosse, E. Kapiteijn, A. M. Kamphuis-Huismans, K. A. T. Naipal, D. Piersma, B. Rikhof, H. M. Westgeest, G. Vreugdenhil, E. Oomen-de Hoop, E. E. A. P. Mulder, Astrid A. M. van der Veldt

**Affiliations:** 1 Department of Medical Oncology and Radiology and Nuclear Medicine, Erasmus Medical Centre Cancer Institute, Dr. Molewaterplein 40, Rotterdam, 3015GD The Netherlands; 2 Department of Surgical Oncology, Erasmus MC Cancer Institute, Rotterdam, The Netherlands; 3 Department of Medical Oncology, Maastricht UMC+, Maastricht, The Netherlands; 4 Department of Medical Oncology, Zuyderland MC, Sittard-Geleen, The Netherlands; 5 Department of Medical Oncology, NKI-AvL, Amsterdam, The Netherlands; 6 Department of Medical Oncology, Radboudumc, Nijmegen, The Netherlands; 7 Department of Medical Oncology, Amsterdam UMC, Vrije Universiteit Amsterdam, Cancer Center Amsterdam, Amsterdam, The Netherlands; 8 Department of Medical Oncology, Isala Zwolle, Zwolle, The Netherlands; 9 Department of Medical Oncology, UMC Groningen, Groningen, The Netherlands; 10 Department of Medical Oncology, Leiden UMC, Leiden, The Netherlands; 11 Department of Medical Oncology, Medisch Spectrum Twente, Enschede, The Netherlands; 12 Department of Medical Oncology, Medisch Centrum Leeuwarden, Leeuwarden, The Netherlands; 13 Department of Medical Oncology, UMC Utrecht, Utrecht, The Netherlands; 14 Department of Medical Oncology, Amphia Ziekenhuis, Breda, The Netherlands; 15 Department of Medical Oncology, Maxima Medisch Centrum Veldhoven, Veldhoven, The Netherlands; 16 Department of Radiology and Nuclear Medicine, Erasmus MC, Rotterdam, The Netherlands

**Keywords:** Melanoma, Ipilimumab, Nivolumab, Discontinuation of treatment, Immune checkpoint inhibitors, Treatment duration

## Abstract

**Background:**

Patients with irresectable stage III or metastatic melanoma presenting with poor prognostic factors are usually treated with a combination of immune checkpoint inhibitors (ICIs), consisting of ipilimumab and nivolumab. This combination therapy is associated with severe immune related adverse events (irAEs) in about 60% of patients. In current clinical practice, patients are usually treated with ICIs for up to two years or until disease progression or the occurrence of unacceptable AEs. The incidence of irAEs gradually increases with duration of treatment. While durable tumour responses have been observed after early discontinuation of treatment, no consensus has been reached on optimal treatment duration. The objective of the Safe Stop IPI-NIVO trial is to evaluate whether early discontinuation of ICIs is safe in patients with irresectable stage III or metastatic melanoma who are treated with combination therapy.

**Methods:**

The Safe Stop IPI-NIVO trial is a nationwide, multicentre, prospective, single-arm, interventional study in the Netherlands. A total of 80 patients with irresectable stage III or metastatic melanoma who are treated with combination therapy of ipilimumab-nivolumab and have a complete or partial response (CR/PR) according to RECIST v1.1 will be included to early discontinue maintenance therapy with anti-PD-1. The primary endpoint is the rate of ongoing response at 12 months after start of ICI. Secondary endpoints include ongoing response at 24 months, disease control at different time points, melanoma specific and overall survival, the incidence of irAEs and health-related quality of life.

**Discussion:**

From a medical, healthcare and economic perspective, overtreatment should be prevented and shorter treatment duration of ICIs is preferred. If early discontinuation of ICIs is safe for patients who are treated with the combination of ipilimumab-nivolumab, the treatment duration of nivolumab could be shortened in patients with a favourable tumour response.

**Trial registration:**

ClinicalTrials.gov ID NCT05652673, registration date: 08–12-2022.

## Introduction

In the past decade, the introduction of systemic therapy with immune checkpoint inhibitors (ICIs) and targeted therapy (TT) has revolutionized the treatment of advanced and metastatic melanoma and has significantly improved the perspectives of patients with this disease [1–3]. ICIs can block programmed cell death protein-1 (anti-PD-1: e.g. nivolumab or pembrolizumab) or cytotoxic T-lymphocyte-associated protein 4 (anti-CTLA-4: e.g. ipilimumab), thereby enhancing the T cell-mediated immune response [4]. By blocking the pathways that regulate the immune system, ICIs do not only stimulate T-cell mediated tumour lysis, but also increase the activity of the immune system against self-antigens, causing organ inflammation [5]. These immune-related adverse events (irAEs) can be severe, lifelong and often require immunosuppressive therapy [6]. Furthermore, the incidence of irAEs gradually increases with duration of treatment [7].

For the treatment of advanced melanoma, combination therapy with nivolumab and ipilimumab is preferred for patients presenting with poor prognostic factors, such as brain metastases, elevated LDH level and/or rapidly progressive disease [8]. In the pivotal randomized trial with three arms in treatment naïve patients with advanced melanoma, four cycles of combination therapy with ipilimumab and nivolumab, followed by maintenance treatment with nivolumab, was compared to nivolumab plus placebo and ipilimumab plus placebo [9]. Treatment was continued until disease progression, the occurrence of unacceptable AEs, or withdrawal of consent. In treatment naïve patients with irresectable stage III or metastatic melanoma, the combination of nivolumab with ipilimumab resulted in a response rate of 58% [9]. The median overall survival (mOS) for patients treated with combination therapy was 72.1 months [10]. In the Checkmate 069 trial, Hodi et al. showed that of 95 patients treated with the combination therapy, 56 (59%) patients had an objective response (CR/PR). At a median follow up of 2 years, 45 of 56 (80%) patients had an ongoing response [11].

Most patients who are treated with the combination therapy of ipilimumab and nivolumab have irAEs, varying from mild (grade 1–2, ~ 30%) to more severe (grade 3–4, ~ 60%) irAEs [12, 13]. Although rare, these irAEs can even be fatal (grade 5, < 5%). The higher rate of irAEs with the combination therapy is primarily caused by the addition of ipilimumab and usually occurs within six months after the start of treatment [13]. However, late-onset irAEs (> 1 year after start of therapy) have been reported in a subset of patients [14]. Most late-onset irAEs occurred in patients who continue with anti-PD-1 therapy, suggesting that continued exposure to anti-PD-1 increases the risk of late-onset irAEs. Therefore, a shorter treatment duration of maintenance treatment with anti-PD-1 may reduce the risk of (late) irAEs, without affecting efficacy [3].

In current clinical practice in the Netherlands, treatment is usually continued for up to a maximum duration of two years or until unacceptable irAEs or disease progression occurs. However, real-world outcomes in the Netherlands showed that treatment duration was often shorter than two years [15], because irAEs resulted in early discontinuation in ~ 50% of all cases. Another real-world study suggested that it is safe to discontinue anti-PD-1 earlier than the protocolized 2 years, regardless of the reason for discontinuation [16]. However, the optimal duration of maintenance treatment with nivolumab remains to be determined prospectively [17]. A shorter treatment duration would yield several major advantages since the current treatment schedule has a high impact on patients in terms of quality of life, healthcare resources and healthcare costs.

Previously, the first nationwide Safe Stop Trial in the Netherlands was initiated for patients with irresectable stage III or metastatic melanoma who were treated with anti-PD-1 monotherapy [18]. The design of this trial is comparable to the current Safe Stop IPI-NIVO Trial. The accrual for the first Safe Stop Trial has been completed successfully: the target number of 200 inclusions has been achieved and the study was considered safe according to the Data Safety Monitoring Board (DSMB). While the results of the first Safe Stop Trial are awaited, this study has shown that patients and physicians are willing to early discontinue ICI and has fuelled the demand for a similar trial for patients who are treated with ipilimumab-nivolumab combination therapy. This nationwide prospective interventional study investigates the safety of early discontinuation of maintenance treatment with anti-PD-1 in patients with irresectable stage III or metastatic melanoma who are treated with combination therapy of ipilimumab and nivolumab.

## Methods

### Primary objective

To assess the rate of ongoing response at 12 months after start of treatment in patients with advanced or metastatic melanoma who are treated with first-line ipilimumab and nivolumab and who discontinue treatment early upon achieving a (confirmed) CR or PR according to RECIST v1.1 [19]. Ongoing response is defined as ongoing complete response (CR) or partial response (PR) according to RECIST v1.1, without disease progression or melanoma-specific mortality. Death due to AEs without evidence of disease progression will not be considered progression.

### Secondary objectives

- To assess ongoing response at 24 months after start of first-line treatment with ipilimumab-nivolumab
- To assess disease control (CR/PR) at the time of the response evaluation
- To assess duration of response (CR/PR) measured until progressive/recurrent disease or melanoma related death, whichever comes first
- To determine melanoma specific survival measured from start of first-line treatment with ipilimumab-nivolumab until melanoma related death
- To determine overall survival measured from start of first-line treatment with ipilimumab-nivolumab until death by any cause
- To assess the need and feasibility of restarting (systemic) treatment for melanoma after progression
- To assess disease control after restarting (systemic) treatment for melanoma
- To assess impact of discontinuation of treatment on irAEs
- To assess impact of discontinuation of treatment on health related quality of life (HRQoL)
- To assess the impact of early discontinuation of treatment on productivity, healthcare resources and hours of informal care

### Study design

**Fig. 1:**
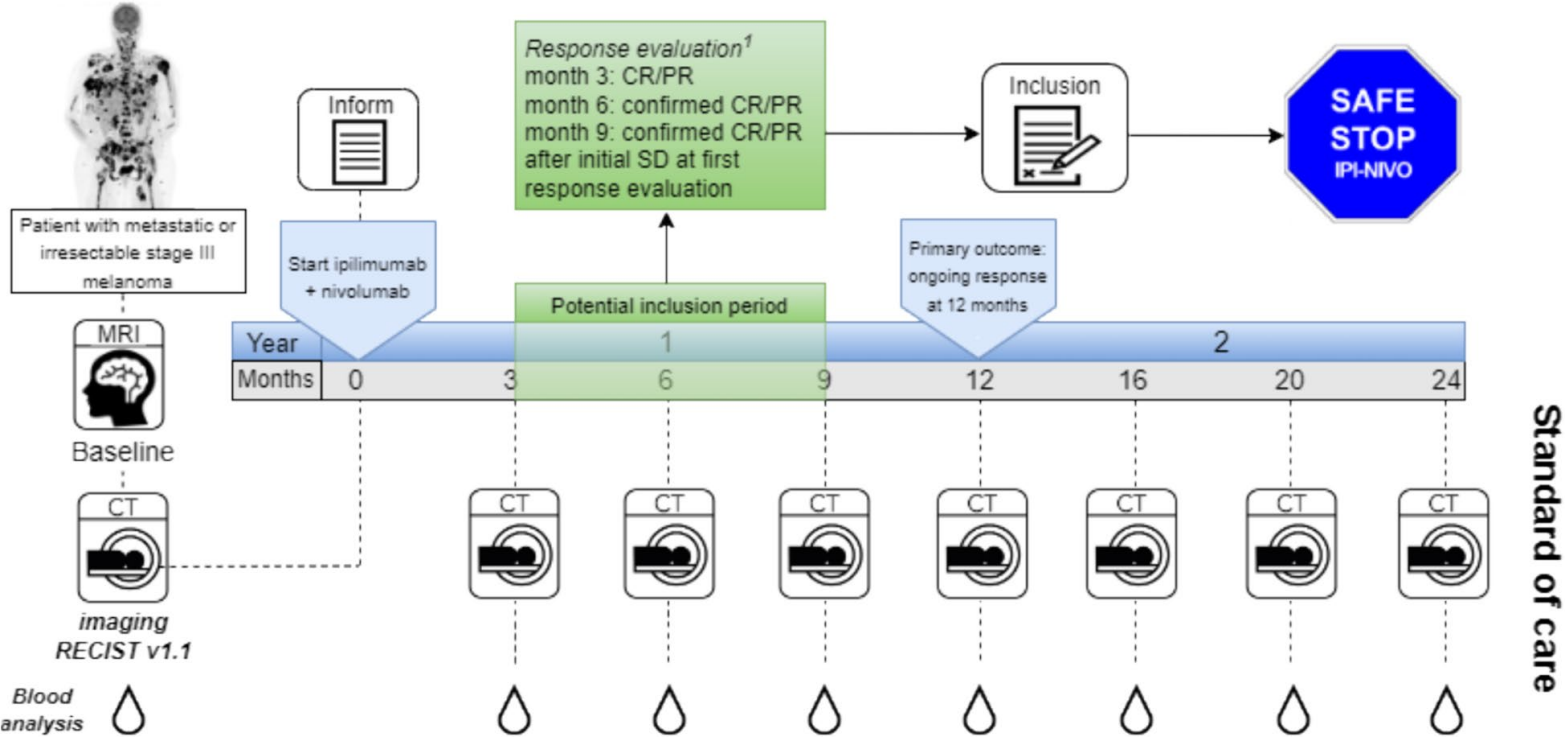
Design of the Safe Stop IPI-NIVO trial. Patients can be included at (confirmed) response, between three and nine months after start of treatment with ipilimumab and nivolumab. Follow-up will be conducted according to standard of care. ^1^ Response evaluation according to RECIST v1.1 [19]. Abbreviations: CR/PR = complete response/partial response, SD = stable disease

This is a nationwide, multicentre, prospective, single-arm intervention study in the Netherlands. Patients with irresectable stage III or metastatic melanoma who are treated with ipilimumab-nivolumab will early discontinue maintenance treatment with anti-PD-1 upon achieving a CR/PR or confirmed CR/PR according to RECIST v1.1. Figure 1 shows a schematic overview of the study design with the potential inclusion period.

In the Netherlands, all patients with advanced or metastatic melanoma are treated in one of the 14 designated Dutch melanoma centres. The current nationwide study will be executed in all 14 centres: Amphia Hospital, Breda; Amsterdam University Medical Centres – location VU, Amsterdam; Antoni van Leeuwenhoek—Netherlands Cancer Institute, Amsterdam; Erasmus Medical Centre Cancer Institute, Rotterdam; Isala Clinics, Zwolle; Leiden University Medical Centre, Leiden; Maastricht University Medical Centre + , Maastricht; Máxima Medical Centre, Veldhoven; Medical Centre Leeuwarden, Leeuwarden; Medical Spectrum Twente, Enschede; Radboud University Medical Centre, Nijmegen; University Medical Centre Groningen, Groningen; University Medical Centre Utrecht, Utrecht; Zuyderland Medical Centre, Sittard-Geleen. In February 2023, the first site (Erasmus Medical Centre Cancer Institute) opened for inclusion.

### Study population

To be eligible to participate in this study, a patient must meet all of the following criteria:

- Age ≥ 18 years
- Irresectable stage III or metastatic melanoma
- At least one cycle of ipilimumab-nivolumab for irresectable stage III or metastatic melanoma and considered to be a candidate for maintenance treatment with anti-PD-1;
- Previous systemic treatment, including immunotherapy, in the (neo)adjuvant setting is allowed;
- Response evaluation according to RECIST v1.1 using a diagnostic CT documenting target lesions every 12 (-2/ + 6) weeks from the start of ipilimumab-nivolumab:
  - ◦ for patients with CR on a diagnostic CT at response evaluation, a low-dose CT (which is usually part of ^18^FDG-PET/CT) is allowed at baseline
  - ◦ for patients with PR on a diagnostic CT at response evaluation, a low-dose CT (which is usually part of.^18^FDG-PET/CT) is allowed if sufficient target lesions are measurable for response evaluation according to RECIST v1.1 criteria
- In case of asymptomatic brain metastases prior to start of first-line ipilimumab-nivolumab, intracerebral tumour response should be confirmed using an MRI for response evaluation prior to inclusion in this study.
- Patients should be included after first CR/PR or first confirmed CR/PR according to RECIST v1.1
- Inclusion should take place no later than 5 weeks after first confirmed CR/PR
- In case of SD at first response evaluation, confirmed CR/PR is required for inclusion
- Eligible and willing to discontinue anti-PD-1 therapy within 4(+ 1) weeks after inclusion, i.e. first CR/PR or first confirmed CR/PR
- Inclusion not later than nine months after start of treatment with ipilimumab-nivolumab
- Presence of MRI of the brain to screen for brain metastasis (prior to discontinuation of ipilimumab-nivolumab)
- Participants with previously locally treated brain metastases are eligible in case they meet the following criteria:
  - ◦ completely asymptomatic brain metastases at inclusion; an incidental epileptic seizure caused by a brain lesion is not considered an exclusion criterion
  - ◦ MRI of the brain at baseline and for response evaluation during treatment

### Follow-up

After inclusion, follow-up will be conducted according to standard of care in the Netherlands, as shown in Table 1. In the first year of follow-up, patients will visit the outpatient clinic every 12 weeks. The visits will be combined with laboratory measurements, diagnostic CT-scan for response evaluation and additional questionnaires for the quality of life. Data will be collected using the Functional Assessment of Cancer Therapy Melanoma (FACT-M), the EuroQoL Health Utilities Index (EQ-5D version 5L), the Cancer Worry Scale (CWS) and the institute for Medical Technology Assessment (iMTA) Resource Use Questionnaire Melanoma.

**Table 1:** Follow-up scheme after first CR/PR first or first confirmed CR/PR

	**Year 1**	**Year 2**	**Year 3 and 4**	**Year 5**
**Visit outpatient clinic**	At inclusion Every 12 weeks	Every 4 months	Every 6 months	Every 12 months
**Laboratory measurements**	At inclusion Every 12 weeks	Every 4 months	Every 6 months	Every 12 months
**Response evaluation (RECIST v1.1)**	At inclusion Every 12 weeks	Every 4 months	Every 6 months	Every 12 months
**Questionnaires**	At inclusion Every 12 weeks	Every 4 months	Every 6 months	Every 12 months

### Statistics primary endpoint

The rate of ongoing response at 12 months is the primary endpoint and will be estimated using the Kaplan–Meier method and its two-sided 95% confidence interval (CI). The study will be declared positive for the primary endpoint if the lower bound of the CI is higher than 82%. A Kaplan Meier plot will be generated to illustrate ongoing response over time. This analysis will not include patients who are lost to follow-up or whose death (without progression) was not melanoma related within the 12 months after start of treatment with ipilimumab and nivolumab. These patients will not be censored, but will be excluded from the analysis for the primary endpoint. For other endpoints, data from these patients will be included in the analyses when statistically feasible. Patients who are lost to follow-up are taken into account in the sample size calculation.

### Sample size calculation

Sample size calculation was performed for the primary endpoint of ongoing response at 12 months. The sample size calculation of this trial is based on the results of the Checkmate-069 study [11] since this prospective study provides detailed patient-level data of first response and duration of response of patients who are treated with first-line combination therapy with ipilimumab-nivolumab. In the Checkmate-069, an ongoing response at 12 months was reported in 92% of patients who had a response within 3 months after start of treatment and were treated for at least 3 months. The hypothesis of the current trial is that the percentage of patients with an ongoing response at 12 months after start of treatment should not be more than 10% worse than the 92% reported in the Checkmate-069. Hence, the null-hypothesis is an ongoing response rate of 82%. Since the rate of ongoing response in the Checkmate-069 is based on a small subgroup, we assume that 90% (instead of 92%) of patients will have an ongoing response at 12 months after start of treatment as input for the alternative hypothesis.

For the sample size calculation, the one-sample log-rank test was used [20]. Given a one-sided type I error of 2.5%, 80% power, a null hypothesis of 82% ongoing response at 12 months, and an alternative hypothesis of 90% ongoing response at 12 months, a total of 20 events (i.e. patients with disease progression) are required. Given an inclusion period of 2 years and 1 year of additional follow-up, 77 patients need to be included to reach this number of events within a total study duration of 3 years. Taking a safety margin of < 5% (*n* = 3 patients) for loss to follow-up or not-melanoma related deaths, the total sample size was set at 80 patients.

### Risk analysis

Patients will be treated and evaluated according to the standard of care in the Netherlands. Since treatment will be discontinued earlier than two years, participation in this trial may affect treatment efficacy, which will be evaluated as the primary objective of this study. To monitor quality and patient safety, a DSMB will be installed. The DSMB consists of a chairman (medical specialist), a medical oncologist and a statistician who are not involved in the study. As early discontinuation of anti-PD-1 should neither lead to an increased mortality or an increased incidence of symptomatic brain metastases, mortality due to disease progression (not toxicity) and newly diagnosed symptomatic brain metastases are considered serious adverse events (SAEs), which will be made available to the DSMB by the study coordinators. Taking into account up-to-date literature, the DSMB will review the data, report their findings to the principal investigator and advise on study continuation after 30 patients are included (with sufficient follow-up of at least 3 months since last inclusion). If the available data after 30 patients are not mature enough (e.g. as a result of fast inclusion), DSMB evaluations will be added until the DSMB has confirmed that the provided data are mature or until 80 patients are included. The principal investigator will submit these reports to the ethics committee along with all relevant data.

## Discussion

This nationwide trial aims to determine the safety and efficacy of early discontinuation of maintenance treatment with anti-PD-1 upon achieving a (confirmed) response for patients with irresectable stage III or metastatic melanoma who are treated with the combination therapy of ipilimumab and nivolumab. The primary endpoint of the trial is the rate of ongoing response, which will be assessed at 12 months after initial start of first-line combination therapy with ipilimumab and nivolumab. To evaluate patients’ QoL, HRQoL questionnaires will be collected periodically.

To ensure feasibility and nationwide implementation, the Safe Stop trials are designed in accordance with current clinical practice in the Netherlands. The timing of scans and laboratory tests are adapted to the current follow-up schedule in daily clinical practice. Therefore, the low-dose CT with FDG-PET is allowed for baseline evaluation under strict pre-specified conditions. In addition, pembrolizumab is allowed as maintenance treatment since pembrolizumab and nivolumab are considered interchangeable based on their similar mechanism of action and survival outcomes [3, 12].

In the current era of rising healthcare costs and scarcity of healthcare resources, efficient use of drugs and resources is of utmost importance [21]. By discontinuing treatment early, costs of expensive drugs, costs of outpatient treatment and -personnel, and potential costs for the treatment of irAEs can be reduced significantly. Furthermore, by minimalizing the burden of hospital visits and potentially preventing irAEs, an increase in patient reported health-related quality of life (HRQoL) can be expected [22].

After succesfull completion of accrual of the first Safe Stop Trial with monotherapy of anti-PD-1 [18], the Safe Stop IPI-NIVO Trial is now open for accrual. Potential advantage of early discontinuation of treatment is the prevention of overtreatment, thereby limiting irAEs and improving HRQoL for patients.

### Trial status

In June 2023, the first patient was included in the study. In April 2024, 19 patients were included and 12 of the 14 Dutch melanoma centres were open for inclusion. Two additional sites are expected to be opened in 2024.

## Data Availability

No datasets were generated or analysed during the current study.

## Abbreviations

AE: Adverse Event
AR: Adverse Reaction
CI: Confidence interval
CR: Complete response
CWS: Cancer Worry Scale
CT: Computed tomography
CTCAE: Common Terminology Criteria for Adverse Events
CTLA-4: Cytotoxic T-lymfocyte-associated protein 4
DMSCG: Dutch Melanoma and Skin Cancer Group
DSMB: Data Safety Monitoring Board
Ecrf: Electronic Case Report Form
EQ-5D-5L: EuroQoL Health Utilities Index (EQ-5D, version 5L)
EudraCT: European drug regulatory affairs Clinical Trials
FACT-M: Functional Assessment of Cancer Therapy Melanoma
FDG-PET: Fluorodeoxyglucose-positron emission tomography
IC: Informed Consent
ICH-GCP: International Conference on Harmonisation Good Clinical Practice
irAEs: Immune-related adverse events
METC: Medical research ethics committee (MREC); in Dutch: medisch-ethische toetsingscommissie (METC)
MRI: Magnetic Resonance Imaging
NFU: Nederlandse Federatie van Universitair Medische Centra (Duch Federation of University Medical Centres)
RECIST v1.1: Response evaluation criteria in solid tumours version 1.1
PD: Progressive disease
PD-1: Programmed cell death protein 1
PR: Partial response
(HR)QoL: (Health related) quality of life
(S)AE: (Serious) Adverse Event
SD: Stable disease
Sponsor: The sponsor is the party that commissions the organisation or performance of the research, for example a pharmaceutical company, academic hospital, scientific organisation or investigator. A party that provides funding for a study but does not commission it is not regarded as the sponsor, but referred to as a subsidising party
SUSAR: Suspected Unexpected Serious Adverse Reaction
WMO: Medical Research Involving Human Subjects Act; in Dutch: Wet Medisch-wetenschappelijk Onderzoek met Mensen
